# Pernicious anaemia and cancer risk in Denmark.

**DOI:** 10.1038/bjc.1996.195

**Published:** 1996-04

**Authors:** L. Mellemkjaer, G. Gridley, H. Møller, A. W. Hsing, M. S. Linet, L. A. Brinton, J. H. Olsen

**Affiliations:** Danish Cancer Society, Division for Cancer Epidemiology, Copenhagen O, Denmark.

## Abstract

A cohort of 5072 patients with pernicious anaemia was identified in the Danish Hospital Discharge Register from 1977 to 1989 and, through linkage to the Danish Cancer Registry, the occurrence of cancer in the cohort was determined up to 1991. Observed numbers of cancer cases during 1-15 years of follow-up were compared with expected numbers based on national incidence rates. Besides the well-established increased risk for stomach cancer, the analysis also revealed a 2-fold increase in the relative risk for cancer of the buccal cavity and pharynx among pernicious anaemia patients in accordance with previous studies; previously reported elevated risks for other digestive tract cancers were not confirmed. There was a non-significantly increased risk for lymphatic and haematological malignancy but the risk tended to disappear after 5 years of follow-up, indicating a possible selection bias. Decreased risks for cervical cancer and non-melanoma skin cancer were also seen.


British Journal of Cancer (1996) 73, 998-1000
© 1996 Stockton Press All rights reserved 0007-0920/96 $12.00

### Pernicious anaemia and cancer risk in Denmark

L Mellemkjaer[1], G Gridley[2], H Møller[3], AW Hsing[2], MS Linet[2], LA Brinton[2] and JH Olsen[1]

[1]Danish Cancer Society, Division for Cancer Epidemiology, Strandboulevarden 49, DK-2100 Copenhagen Ø, Denmark; [2]Epidemiology and Biostatistics Program, Division of Cancer Etiology, National Cancer Institute, Bethesda, Maryland 20892, USA; [3]Centre for Research in Health and Social Statistics, Sejrøgade 11, DK-2100 Copenhagen Ø, Denmark.

**Summary** A cohort of 5072 patients with pernicious anaemia was identified in the Danish Hospital Discharge Register from 1977 to 1989 and, through linkage to the Danish Cancer Registry, the occurrence of cancer in the cohort was determined up to 1991. Observed numbers of cancer cases during 1–15 years of follow-up were compared with expected numbers based on national incidence rates. Besides the well-established increased risk for stomach cancer, the analysis also revealed a 2-fold increase in the relative risk for cancer of the buccal cavity and pharynx among pernicious anaemia patients in accordance with previous studies; previously reported elevated risks for other digestive tract cancers were not confirmed. There was a non-significantly increased risk for lymphatic and haematological malignancy but the risk tended to disappear after 5 years of follow-up, indicating a possible selection bias. Decreased risks for cervical cancer and non-melanoma skin cancer were also seen.

**Keywords:** pernicious anaemia; cancer risk; cohort study

Several previous studies have documented an increased incidence of stomach cancer in patients with pernicious anaemia (PA) (Blackburn *et al.*, 1968; Elsborg and Mosbech, 1979; Borch *et al.*, 1988; Brinton *et al.*, 1989; Hsing *et al.*, 1993). An increase in the risk for other upper gastrointestinal cancers such as buccal cavity and pharynx (Brinton *et al.*, 1989; Hsing *et al.*, 1993) and oesophagus (Hsing *et al.*, 1993) has also been reported, as well as increases for other digestive organ cancers [liver, biliary tract and pancreas (Hsing *et al.*, 1993)], though these cancers have been investigated less extensively among PA patients than stomach cancer. Of possible relevance to the autoimmune process in PA, studies in the US (Brinton *et al.*, 1989) and Sweden (Hsing *et al.*, 1993) have also reported increased risks for multiple myeloma and myeloid leukaemia. We have performed a linkage study similar in size to the large US and Swedish studies between the nationwide Hospital Discharge and Cancer Registries in Denmark in order to clarify further the risk for gastrointestinal and other cancers among patients with PA.

### Materials and methods

Since 1977 the Hospital Discharge Register has kept records of more than 99% of all discharges from non-psychiatric hospitals in Denmark (Danish National Board of Health, 1981). For each discharge the register includes a personal identification number unique to every Danish citizen, date of discharge, up to 20 discharge diagnoses coded according to a modified version of ICD-8 (Danish National Board of Health, 1976) and codes for surgical procedures carried out during the hospitalisation (Danish National Board of Health, 1973). All 6194 patients with ICD-8 codes 281.00–09 notified in the Hospital Discharge Register during 1977–89 were linked by the personal identification number to the Central Population Register, whereby the personal number was verified and dates of death or emigration were obtained. In this process, 11 (0.2%) patients were excluded because either they were not Danish residents or had an invalid identification number.

For each patient, all discharges before and after the first discharge with PA were also identified from the register file to provide a full hospitalisation history covering the period 1977–89. Detailed evaluation of the hospitalisation history led to identification of 43 (0.7%) patients who had undergone resection of the stomach. Since this surgical procedure may lead to deficiency of intrinsic factor and thereby to secondary PA, these patients were excluded from the study. Follow-up started one year after the first known hospital discharge reporting PA and continued until date of death, date of emigration or the end of December 1991, whichever came first. Excluding the first year of follow-up meant that 1068 (17%) patients who died on or within one year from the PA hospitalisation did not contribute any follow-up time and they were not counted in the final study cohort of 5072 patients. Those patients with PA who subsequently developed cancer were ascertained through the linkage to the Danish Cancer Registry (Storm *et al.*, 1994). The expected number of cancers was calculated from accumulated person–years and national incidence rates, sub-divided by sex, age and calendar time in 5 year intervals. Statistical methods used were based on the assumption that the observed number of cancer cases follows a Poisson distribution. Confidence intervals for the relative risk (RR), i.e. the ratio of observed to expected cancers, were computed using exact Poisson limits when the observed number of cases was less than 10; otherwise Byar's approximation was used (Rothman and Boice, 1979).

### Results

During the follow-up of 5072 PA patients, 25 768 person-years were accrued with a mean follow-up interval of 5.1 years (range 1–15 years). The mean age at entry to the study was 71 years for men and 73 years for women. The majority of patients were women (66%).

The overall number of neoplasms observed during 1–15 years of follow-up was in accordance with the expected number (Table I). A two-fold excess of stomach cancer was seen, including one patient with a carcinoid tumour. In addition, there was a significant increase in the risk for cancer of the buccal cavity and pharynx. Risks were increased among both men and women for these cancers. The excess of cancer of the buccal cavity and pharynx was not confined to any specific subsite such as lip, tongue or salivary glands. One patient with cancer of the mouth and one with cancer of the tonsil had a diagnosis of alcoholism in the Hospital Discharge Register before the cancer diagnosis. Women

Correspondence: L Mellemkjaer
Received 6 September 1995; revised 6 November 1995; accepted 6 November 1995

experienced a significantly increased risk for oesophageal cancer whereas men experienced a reduced risk. The risk for non-Hodgkin's lymphoma, multiple myeloma and non-lymphocytic leukaemia was moderately but not significantly increased. Significantly reduced risks were observed for cervical cancer and non-melanoma skin cancer (Table I).

The elevated RRs for cancer of the stomach and buccal cavity and pharynx were comparable in the two periods of follow-up, 1–4 and 5–15 years (Table II). Excesses of non-Hodgkin's lymphoma and non-lymphocytic leukaemia were confined to the period 1–4 years of follow-up, whereas there was a small excess of multiple myeloma during both early and late follow-up.

### Discussion

In this cohort study of patients with PA we found a two-fold increase in the risk for stomach cancer in accordance with previous estimates (Blackburn *et al.*, 1968; Brinton *et al.*, 1989; Hsing *et al.*, 1993). Our finding of an excess of cancer of the buccal cavity and pharynx is consistent with findings in the two large US and Swedish studies (Brinton *et al.*, 1989; Hsing *et al.*, 1993). We have no information on important risk factors such as alcohol intake and smoking habits but two cases of buccal cavity and pharynx cancer had mention of alcoholism in the hospital record. There was, however, no general excess of other alcohol- or tobacco-related cancers in

**Table 1** Observed (Obs) numbers and relative risks (RR) of cancer among patients with pernicious anaemia (PA) followed 1–15 years after the hospitalisation with PA

| | Both sexes | | | | Men | | | Women | | |
|---|---|---|---|---|---|---|---|---|---|---|---|---|
| *Cancer site* | *Obs* | *Exp*[a] | *RR* | *95% CI*[b] | *Obs* | *RR* | *95% CI* | *Obs* | *RR* | *95% CI* |
| All malignant neoplasms | 497 | 495.2 | 1.00 | 0.92–1.10 | 220 | 1.05 | 0.92–1.20 | 277 | 0.97 | 0.86–1.09 |
| Buccal cavity and pharynx | 16 | 8.0 | 2.0 | 1.1–3.3 | 8 | 1.8 | 0.8–3.5 | 8 | 2.3 | 1.0–4.5 |
| Lip | 2 | 2.4 | 0.8 | 0.1–3.1 | 0 | – | – | 2 | 4.4 | 0.5–15.9 |
| Tongue | 2 | 1.0 | 2.0 | 0.2–7.3 | 1 | 2.7 | 0.1–15.0 | 1 | 1.6 | 0.0–9.0 |
| Salivary glands | 0 | 0.8 | – | – | 0 | – | – | 0 | – | – |
| Mouth | 7 | 2.4 | 2.9 | 1.2–6.0 | 3 | 3.1 | 0.6–9.2 | 4 | 2.8 | 0.8–7.1 |
| Pharynx | 5 | 1.5 | 3.4 | 1.1–7.9 | 4 | 4.5 | 1.2–11.4 | 1 | 1.7 | |
| Oesophagus | 8 | 4.6 | 1.7 | 0.7–3.4 | 1 | 0.4 | 0.0–2.4 | 7 | 3.1 | 1.2–6.4 |
| Stomach | 50 | 21.2 | 2.4 | 1.7–3.1 | 26 | 2.7 | 1.7–3.9 | 24 | 2.1 | 1.3–3.1 |
| Small intestine | 2 | 1.3 | 1.5 | 0.2–5.5 | 1 | 1.8 | 0.0–10.0 | 1 | 1.3 | 0.0–7.3 |
| Colon | 50 | 52.6 | 1.0 | 0.7–1.3 | 17 | 1.0 | 0.6–1.5 | 33 | 1.0 | 0.7–1.3 |
| Rectum | 19 | 25.3 | 0.8 | 0.5–1.2 | 11 | 1.0 | 0.5–1.7 | 8 | 0.6 | 0.3–1.1 |
| Liver | 3 | 5.2 | 0.6 | 0.1–1.7 | 3 | 1.2 | 0.3–3.6 | 0 | – | – |
| Gallbladder and biliary tract | 6 | 6.6 | 0.9 | 0.3–2.0 | 3 | 2.0 | 0.4–5.8 | 3 | 0.6 | 0.1–1.7 |
| Pancreas | 19 | 17.2 | 1.1 | 0.7–1.7 | 7 | 1.1 | 0.4–2.3 | 12 | 1.1 | 0.6–1.9 |
| Lung | 45 | 49.4 | 0.9 | 0.7–1.2 | 28 | 0.9 | 0.6–1.3 | 17 | 1.0 | 0.6–1.6 |
| Breast | 39 | 48.4 | 0.8 | 0.6–1.1 | 1 | 2.9 | 0.1–16.1 | 38 | 0.8 | 0.6–1.1 |
| Cervix uteri | 1 | 5.9 | 0.2 | 0.0–0.9 | – | – | – | 1 | 0.2 | 0.0–0.9 |
| Corpus uteri | 10 | 10.4 | 1.0 | 0.5–1.8 | – | – | – | 10 | 1.0 | 0.5–1.8 |
| Ovary | 6 | 10.0 | 0.6 | 0.2–1.3 | – | – | – | 6 | 0.6 | 0.2–1.3 |
| Prostate | 39 | 33.4 | 1.2 | 0.8–1.6 | 39 | 1.2 | 0.8–1.6 | – | – | – |
| Kidney | 14 | 12.7 | 1.1 | 0.6–1.8 | 6 | 1.1 | 0.4–2.3 | 8 | 1.1 | 0.5–2.2 |
| Urinary bladder | 23 | 28.1 | 0.8 | 0.5–1.2 | 16 | 0.9 | 0.5–1.4 | 7 | 0.7 | 0.3–1.5 |
| Melanoma | 4 | 6.9 | 0.6 | 0.2–1.5 | 1 | 0.4 | 0.0–2.5 | 3 | 0.7 | 0.1–1.9 |
| Non-melanoma skin cancer | 57 | 74.4 | 0.8 | 0.6–1.0 | 19 | 0.6 | 0.4–1.0 | 38 | 0.9 | 0.6–1.2 |
| Brain and nervous system | 7 | 6.7 | 1.0 | 0.4–2.2 | 3 | 1.3 | 0.3–3.8 | 4 | 0.9 | 0.2–2.3 |
| Lymphatic and haematological | 35 | 28.4 | 1.2 | 0.9–1.7 | 15 | 1.2 | 0.7–2.0 | 20 | 1.2 | 0.8–1.9 |
| Non-Hodgkin's lymphoma | 14 | 9.6 | 1.5 | 0.8–2.5 | 3 | 0.8 | 0.2–2.4 | 11 | 1.9 | 0.9–3.3 |
| Multiple myeloma | 7 | 5.3 | 1.3 | 0.5–2.7 | 3 | 1.3 | 0.3–3.8 | 4 | 1.3 | 0.4–3.4 |
| Leukaemia | 13 | 12.5 | 1.0 | 0.6–1.8 | 8 | 1.4 | 0.6–2.8 | 5 | 0.7 | 0.2–1.7 |
| Non-lymphocytic leukaemia[c] | 9[d] | 5.2 | 1.7 | 0.8–3.3 | 4 | 1.8 | 0.5–4.6 | 5 | 1.7 | 0.6–4.0 |
| Other specified sites | 26 | 21.7 | 1.2 | 0.8–1.8 | 8 | 0.9 | 0.4–1.8 | 18 | 1.4 | 0.8–2.2 |
| Secondary and unspecified sites | 18 | 17.9 | 1.1 | 0.6–1.7 | 7 | 1.2 | 0.5–2.5 | 11 | 1.0 | 0.5–1.8 |

[a] Expected numbers of cancer. [b] Confidence interval. [c]Non-lymphocytic leukaemia includes chronic and acute myeloid leukaemia, monocytic leukaemia and erythroleukaemia. [d] Three cases of chronic myeloid leukaemia and six cases of acute myeloid leukaemia.

**Table II** Observed (Obs) numbers and relative risks (RR) of cancer for selected sites according to length of follow-up among patients with pernicious anaemia (PA)

| | Length of follow-up (years) | | | | | |
|---|---|---|---|---|---|---|
| | | 1–4 | | | 5–15 | |
| *Person-years* | | 15429 | | | 10339 | |
| *Cancer site* | *Obs* | *RR* | *95% CI*[a] | *Obs* | *RR* | *95% CI* |
| Buccal cavity and pharynx | 10 | 2.1 | 1.0–3.8 | 6 | 1.9 | 0.7–4.2 |
| Lip | 2 | 1.4 | 0.2–4.9 | 0 | – | – |
| Tongue | 1 | 1.7 | 0.0–9.4 | 1 | 2.5 | 0.1–14.0 |
| Salivary glands | 0 | – | – | 0 | – | – |
| Mouth | 5 | 3.5 | 1.1–8.1 | 2 | 2.1 | 0.3–7.7 |
| Pharynx | 2 | 2.2 | 0.3–8.1 | 3 | 5.1 | 1.1–15.0 |
| Stomach | 32 | 2.4 | 1.6–3.3 | 18 | 2.4 | 1.4–3.7 |
| Cervix uteri | 1 | 0.3 | 0.0–1.6 | 0 | – | – |
| Non-melanoma skin cancer | 39 | 0.9 | 0.6–1.2 | 18 | 0.6 | 0.3–0.9 |
| Lymphatic and haematological cancers | 25 | 1.5 | 1.0–2.2 | 10 | 0.9 | 0.4–1.6 |
| Non-Hodgkin's lymphoma | 11 | 2.0 | 1.0–3.6 | 3 | 0.7 | 0.2–2.2 |
| Multiple myeloma | 4 | 1.3 | 0.3–3.2 | 3 | 1.4 | 0.3–4.2 |
| Non-lymphocytic leukaemia | 7 | 2.2 | 0.9–4.6 | 2 | 1.0 | 0.1–3.6 |

[a] Confidence interval.

the cohort to suggest that alcohol or tobacco were not important confounders.

Megaloblastic transformation owing to cobalamin deficiency in PA which includes chromosomal changes, is particularly seen in blood cell lines but may also occur in the epithelial cells of the gastrointestinal tract (Babior, 1990). Such changes, however, are not reflected in excess risks of small intestine and haematopoietic cancers comparable to those of buccal cavity, pharynx and stomach cancers. Risks for some lymphatic and haematological cancers were increased but not significantly as in previous investigations (Brinton *et al.*, 1989; Hsing *et al.*, 1993). The reason for the difference between results may be that we, in contrast to the previous studies, excluded the first year of follow-up, where there was an accumulation of non-Hodgkin's lymphoma, multiple myeloma and myeloid leukaemia in our study. This is parallel to observations for multiple myeloma and myeloid leukaemia in the Swedish study (AW Hsing, personal communication) and for myeloid leukaemia in the US study (Brinton *et al.*, 1989). In our opinion, the initial excess of cases is probably due to inclusion of misdiagnosed haematopoietic cancer in the PA cohort and does not reflect a genuine association. Our analysis revealed a deficit of cervical cancer and non-melanoma skin cancer; the latter predominantly among men. These findings may be due to chance although the reduced risk for the non-melanoma is consistent with results from the Swedish study (Hsing *et al.*, 1993).

**Acknowledgements**
We thank Joseph K McLaughlin and Joseph F Fraumeni Jr for useful comments on the manuscript and Andrea Bautz for programming support. We also thank the National Board of Health for access to data from the Hospital Discharge Register. This study was supported by MAO NO1-CP-85639-04 from the National Cancer Institute, Bethesda, Maryland, USA.

### References

BABIOR BM. (1990). The megaloblastic anemias. In *Hematology*, Williams WJ, Beutler E, Erslev AJ and Lichtman MA. (eds) pp. 453–481. McGraw-Hill: New York.

BLACKBURN EK, CALLENDER ST, DACIE JV, DOLL R, GIRDWOOD RH, MOLLIN DL, SARACCI R, STAFFORD JL, THOMPSON RB, VARADI S AND WETHERLEY-MEIN G. (1968). Possible association between pernicious anaemia and leukaemia: a prospective study of 1,625 patients with a note on the very high incidence of stomach cancer. *Int. J. Cancer*, **3**, 163–170.

BORCH K, KULLMAN E, HALLHAGEN S, LEDIN T AND IHSE I. (1988). Increased incidence of pancreatic neoplasia in pernicious anemia. *World. J. Surg.*, **12**, 866–870.

BRINTON LA, GRIDLEY G, HRUBEC Z, HOOVER R AND FRAUMENI JF JR. (1989). Cancer risk following pernicious anaemia. *Br. J. Cancer*, **59**, 810–813.

DANISH NATIONAL BOARD OF HEALTH. (1973). *Classification of Surgical Procedures and Therapies*. (in Danish). Danish National Board of Health: Copenhagen.

DANISH NATIONAL BOARD OF HEALTH. (1976). *Classification of Diseases*. (in Danish). Danish National Board of Health: Copenhagen.

DANISH NATIONAL BOARD OF HEALTH. (1981). *The Activity in the Hospital Care System*. (in Danish). Danish National Board of Health: Copenhagen.

ELSBORG L AND MOSBECH J. (1979). Pernicious anaemia as a risk factor in gastric cancer. *Acta Med. Scand.*, **206**, 315–318.

HSING AW, HANSSON LE, MCLAUGHLIN JK, NYRÉN O, BLOT WJ, EKBOM A AND FRAUMENI JF JR. (1993). Pernicious anemia and subsequent cancer. A population-based cohort study. *Cancer*, **71**, 745–750.

ROTHMAN KJ AND BOICE JD. (1979). *Epidemiologic Analysis with a Programmable Calculator*. DHHS Publication No (NIH) 79-1649). US Government Printing Office: Washington DC.

STORM HH, MANDERS T AND LECKERS S. (1994). *Cancer Incidence in Denmark 1991*. Danish Cancer Society: Copenhagen.


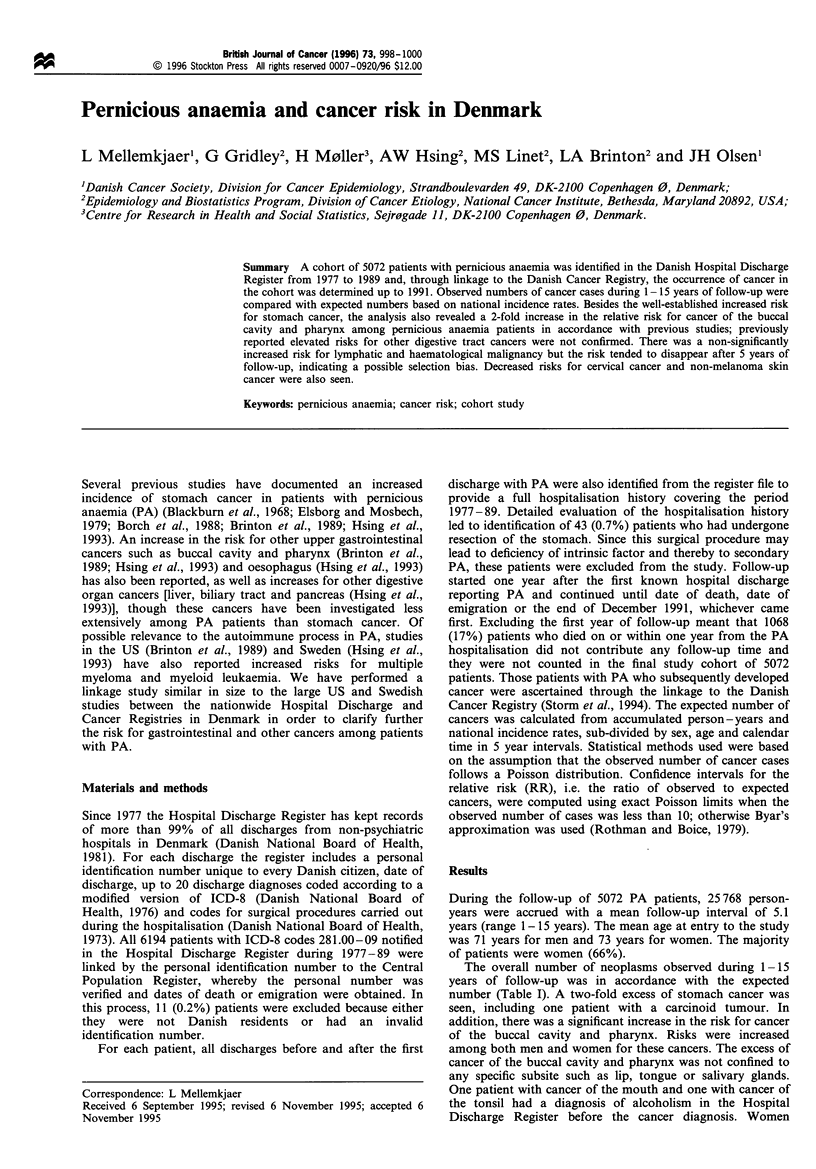

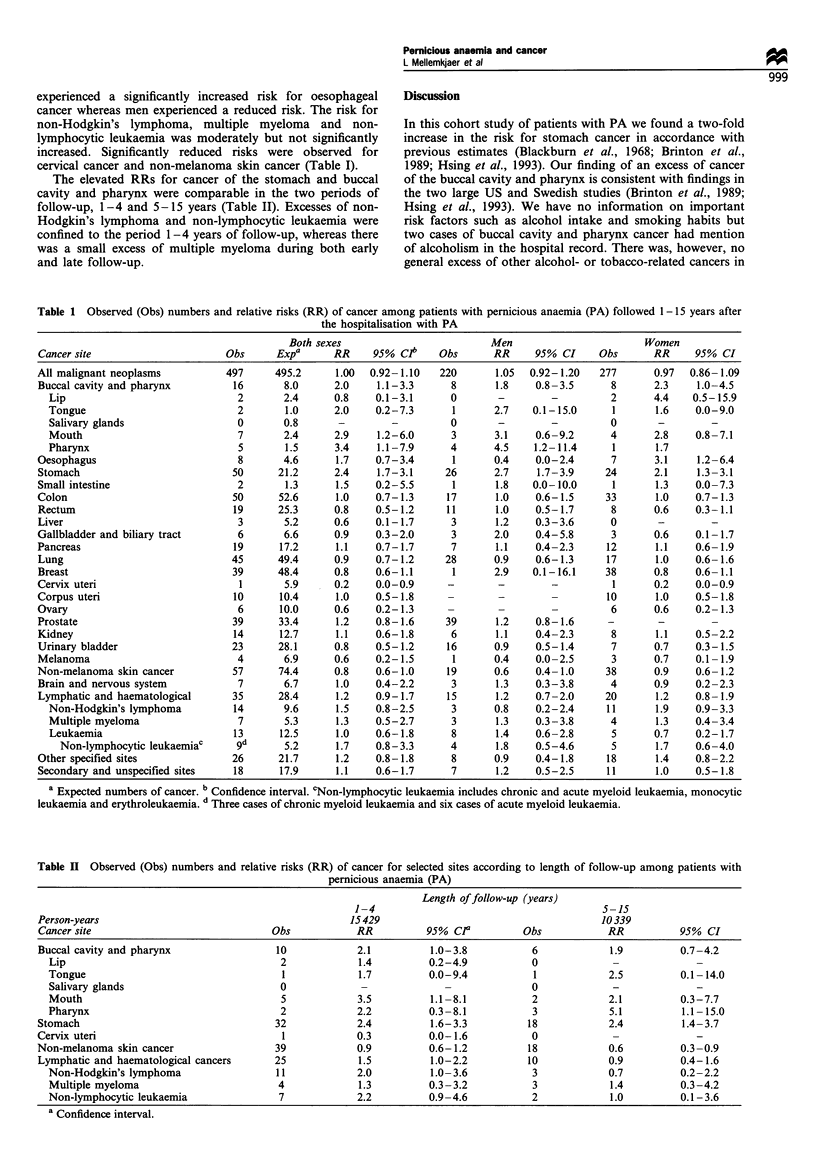

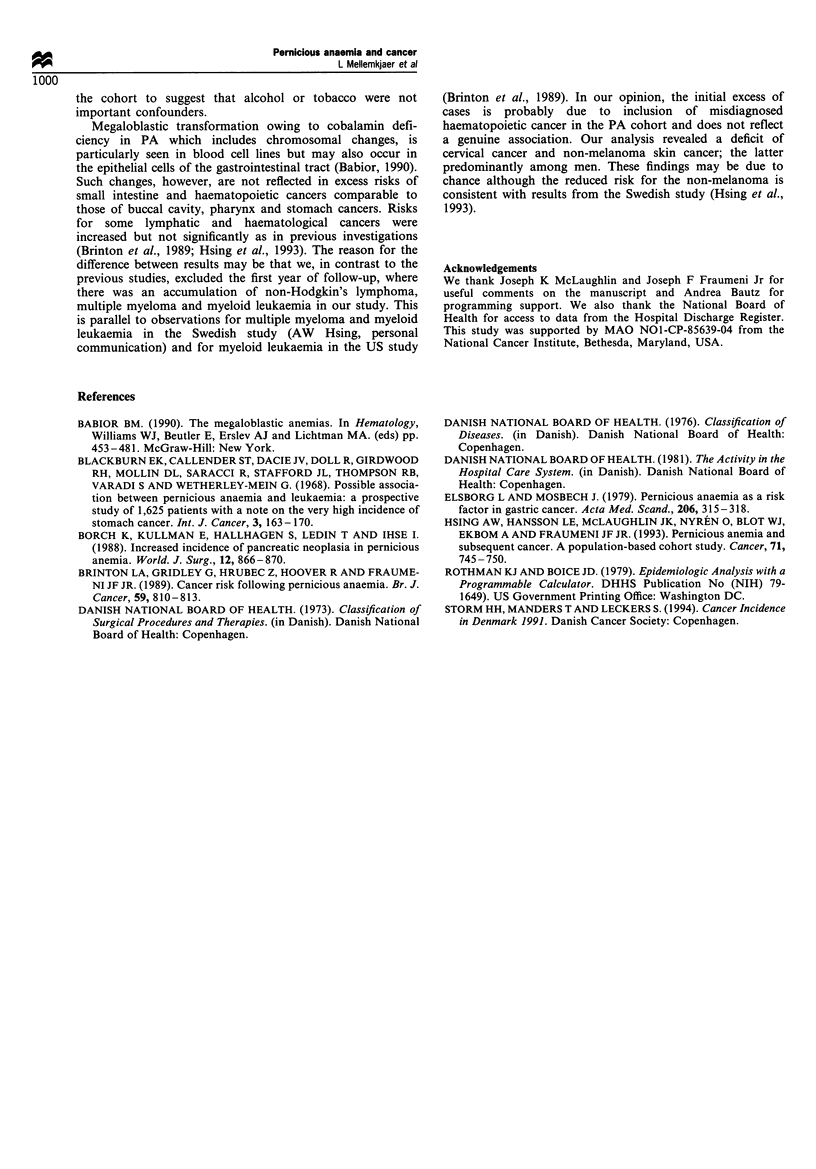


## References

[OCR_00290] Blackburn E. K., Callender S. T., Dacie J. V., Doll R., Girdwood R. H., Mollin D. L., Saracci R., Stafford J. L., Thompson R. B., Varadi S. (1968). Possible association between pernicious anaemia and leukaemia: a prospective study of 1,625 patients with a note on the very high incidence of stomach cancer.. Int J Cancer.

[OCR_00298] Borch K., Kullman E., Hallhagen S., Ledin T., Ihse I. (1988). Increased incidence of pancreatic neoplasia in pernicious anemia.. World J Surg.

[OCR_00303] Brinton L. A., Gridley G., Hrubec Z., Hoover R., Fraumeni J. F. (1989). Cancer risk following pernicious anaemia.. Br J Cancer.

[OCR_00323] Elsborg L., Mosbech J. (1979). Pernicious anaemia as a risk factor in gastric cancer.. Acta Med Scand.

[OCR_00325] Hsing A. W., Hansson L. E., McLaughlin J. K., Nyren O., Blot W. J., Ekbom A., Fraumeni J. F. (1993). Pernicious anemia and subsequent cancer. A population-based cohort study.. Cancer.

